# The Reliability of Electromyographic Normalization Methods for Cycling Analyses

**DOI:** 10.1515/hukin-2015-0030

**Published:** 2015-07-10

**Authors:** Jonathan Sinclair, Paul John Taylor, Jack Hebron, Darrell Brooks, Howard Thomas Hurst, Stephen Atkins

**Affiliations:** 1 Division of Sport, Exercise and Nutritional Sciences University of Central Lancashire.

**Keywords:** electromyography, normalization, rectus femoris, biceps femoris, gastrocnemius, tibialis anterior

## Abstract

Electromyography (EMG) is normalized in relation to a reference maximum voluntary contraction (MVC) value. Different normalization techniques are available but the most reliable method for cycling movements is unknown. This study investigated the reliability of different normalization techniques for cycling analyses. Twenty-five male cyclists (age 24.13 ± 2.79 years, body height 176.22 ± 4.87 cm and body mass 67.23 ± 4.19 kg, BMI = 21.70 ± 2.60 kg·m-1) performed different normalization procedures on two occasions, within the same testing session. The rectus femoris, biceps femoris, gastrocnemius and tibialis anterior muscles were examined. Participants performed isometric normalizations (IMVC) using an isokinetic dynamometer. Five minutes of submaximal cycling (180 W) were also undertaken, allowing the mean (DMA) and peak (PDA) activation from each muscle to serve as reference values. Finally, a 10 s cycling sprint (MxDA) trial was undertaken and the highest activation from each muscle was used as the reference value. Differences between reference EMG amplitude, as a function of normalization technique and time, were examined using repeated measures ANOVAs. The test-retest reliability of each technique was also examined using linear regression, intraclass correlations and Cronbach’s alpha. The results showed that EMG amplitude differed significantly between normalization techniques for all muscles, with the IMVC and MxDA methods demonstrating the highest amplitudes. The highest levels of reliability were observed for the PDA technique for all muscles; therefore, our results support the utilization of this method for cycling analyses.

## Introduction

The analysis of muscle activation using electromyography (EMG) is frequently used to examine a range of athletic and occupational movements (De Luca, 1997; Cram, 2003). It has been recognised that intrinsic and extrinsic factors can cause significant fluctuations in the raw EMG signal, reducing longitudinal reliability and increasing inter and intra subject variability (Kasprisin and Grabiner, 1998). Therefore, contrasting raw EMG magnitude between individuals leads to a misinterpretation of the EMG signal amplitude (Kasprisin and Grabiner, 1998). In order to allow EMG amplitudes to be equitably contrasted between participants, days and muscles, a normalization method is employed. The EMG signal during dynamic activity is normalized in relation to a reference amplitude, typically referred to as a maximum voluntary contraction (MVC) (Burden and Bartlett, 1999; Knutson et al., 1994). Normalization rescales the raw EMG amplitudes from millivolts into a percentage of this reference value (Burden and Bartlett, 1999).

A number of different methods are currently available in the literature to produce reference EMG values for normalization purposes (Burden et al., 2003; Lehman and McGill, 1999). Currently, there is no universally agreed method for normalization of EMG data (Albertus-Kajee et al., 2010) and a variety of techniques are still used interchangeably to produce normalization reference values (Sinclair et al., 2012). Selection of the normalization method is important for the interpretation of the EMG signals’ magnitude (Ball and Scurr, 2011). Whilst within a study EMG values are relative to the normalization amplitude, in the context of other literature they may not be comparable if an alternate normalization method is used (Sinclair et al., 2012).

The most common EMG normalization procedure is to use the reference value derived from the same muscle during a maximal isometric contraction (IMVC). IMVCs have been criticised on the basis that they may not represent the maximum activation capacity of the muscle in situations other than those at which the IMVC was performed. Utilization of IMVCs in clinical research is considered to have little relevance as symptomatic participants are habitually unable to produce maximal isometric muscle actions (Ekstrom et al., 2005). The limitations associated with IMVCs have led to the development of dynamic normalization techniques whereby, either the mean (MDA) or peak (PDA) EMG amplitude within the activity under investigation serves as the reference value. Finally a method has been advocated whereby the same muscle actions as those under investigation are performed only with maximal intensity over short duration (Albertus-Kajee et al., 2010). This procedure is referred to as the sprint technique (MxDA) and the normalization amplitude is representative of the maximum muscle amplitude value obtained during the high intensity movement.

Reliability is the extent to which measurements are consistent, researchers should normalize muscle activity using the most reliable method in order to make appropriate inferences regarding muscle activation (Sinclair et al., 2012). Previous analyses have examined the reliability of the different normalization techniques. Bolgla and Uhl (2007) investigated the reliability of the IMVC, MDA and PDA techniques for the quantification of hip abductor activation. They demonstrated that the IMVC method demonstrated the highest levels of reliability. Netto and Burnett (2004) considered the reliability of maximal/ sub-maximal IMVCs obtained using an isokinetic dynamometer in addition to a manual IMVC against manual resistance for EMG analysis of the neck muscles. It was demonstrated that all methods showed good levels of reliability. Sinclair et al. (2012) investigated the reliability of various normalization methods for the stance phase of running. It was demonstrated that the PDA showed the highest level of reliability whereas the IMVC technique was associated with the lowest levels of reliability. Similarly, Ball and Scurr (2011) demonstrated that IMVCs exhibited the lowest reliability whilst the PDA method was linked with the highest reliability when quantifying high speed muscle actions. There is currently a paucity of reliability data for EMG normalization procedures during cycling. Thus, the most reliable normalization procedure remains unknown for cycling analyses.

The aim of this investigation was firstly to determine the most reliable technique for the normalization of muscle activation during cycling from the relevant methods available within the literature. Secondly, the study aimed to examine whether the different normalization techniques produced different reference amplitudes. This study firstly tested the hypothesis that different normalization techniques would produce different reference EMG amplitudes and secondly that PDA normalization techniques would produce the most reliable MVC amplitudes.

## Material and Methods

### Participants

Twenty-five (age 24.13 ± 2.79 years, body height 176.22 ± 4.87 cm and body mass 67.23 ± 4.19 kg, BMI = 21.70 ± 2.60 kg·m^−1^) male cyclists took part in the current investigation. All were free from lower extremity injury at the time of data collection and provided written informed consent. All participants were currently engaged in cycling training and confirmed that they completed a minimum of 3 rides per week and 300 km per month. The procedure utilized for this investigation was approved by the Ethics Committee of the University of Central Lancashire, in accordance with the Declaration of Helsinki.

### EMG preparation

Surface EMG activity was obtained, at a sampling frequency of 1000 Hz, from the Rectus Femoris (RF), Tibialis Anterior (TA), Gastrocnemius (GM) and Biceps Femoris (BF) muscles. Bipolar electrodes, with an interelectrode distance of 19 mm, connected to an interface unit (Biometrics LTD, SX230FW) were used. All recordings were taken from the right (dominant) side of the body. To minimize cross-talk interference from nearby muscles, the electrodes were placed on the bellies on the appropriate muscles in alignment with the muscle pennation, and sited according to the guidelines of SENIAM (Freriks et al., 1999). Prior to electrode placement, the skin was shaved, abraded then cleaned using an ethanol swab to minimize skin impedance, and support proper recordings of the muscle electrical potentials.

### Isometric MVC (IMVC)

Firstly the isometric measurements of the lower extremity muscles were undertaken using an isokinetic dynamometer (Isocom, Phoenix Healthcare, Nottingham). All isometric MVC values were obtained using this device. Each participant’s thighs and upper body were attached firmly to the dynamometer, and the arms folded across the chest to minimize upper body contribution. For the RF and BF muscles, isometric muscle actions were obtained with the knee sagittal plane angle maintained at 60°. The functional axis of rotation of the knee joint aligned with the axis of rotation of the dynamometer lever arm. The concerned limb was also rigidly secured to the lever arm, just above the malleoli. When measuring EMG output from the GM and TA muscles, the lower leg was elevated and the thigh was positioned on a holder. The foot was rigidly strapped against a foot-plate resulting in a sagittal plane knee angle of 30°. As with the knee joint, the functional axis of rotation of the ankle was aligned with the axis of rotation of the lever arm. The ankle was maintained at an angle of 15° (with 0° being a fully vertical shank) from where the subjects had to either push or pull against the foot-plate using the GM and TA muscles. The isometric test protocol included three repeats of 5 s each separated by 60 s intervals in accordance with Albertus-Kajee et al. (2010). All subjects were encouraged verbally to exert maximal effort during their IMVC trials.

### Cycling procedures

After a rest period of 10 minutes participants progressed onto the cycling analyses. All cycling actions were completed using a cycle ergometer (Monark Ergomedic 874E, Monark Exercise, AB, Varberg, Sweden). For acquisition of the mean dynamic activity (MDA) and peak dynamic activity (PDA) normalization values, participants were required to cycle at a constant workload of 180 W for 5 minutes. Pedal cadence was maintained at 80 rev·min^−1^ throughout. EMG amplitude from the last 10 s of each minute were extracted, resulting in 13.33 ± 0.21 pedal cycles per 10 s, and 66.65 pedal cycles in total. Saddle height was determined using the LeMond (1987)formula. For the acquisition of the sprint method (MxDA) normalization value participants were required to undertake a 10 s maximal sprint whereby participants started the sprint from power output of 180 W.

### Reliability

Following a 30 min rest period the protocol was repeated, providing pre and post values for each normalization technique thus allowing reliability to be assessed. The EMG electrodes were not removed at any point and the saddle height and seating position/ length of leg attachments of the isokinetic dynamometer were not changed between test and retest trials.

#### Data processing

The raw EMG signals (Mv) from each muscle during each technique were full wave rectified and filtered using a 20 Hz Butterworth zero lag low-pass 4^th^ order filter to create a linear envelope. EMG processing and normalization to the pedal cycle were undertaken using Visual 3D (C-Motion, Germantown, MD, USA). Due to the variance in EMG amplitudes that is provided by altering the filtering technique and cut-off frequency, this EMG processing technique was applied to the EMG activity from all normalization methods.

### Normalization techniques

#### Isokinetic (IMVC)

The ensemble average of the peak muscle activation (for each muscle) from each of the three trials was considered to be IMVCa and the highest peak reading of the three trials was considered to be IMVCb.

#### Mean dynamic activity (MDA)

The ensemble average of the mean activation during the pedal cycle (for each muscle) was considered to be MDA.

#### Peak dynamic activity (PDA)

The ensemble average of the peak activation during the pedal cycle (for each muscle) was considered to be PDAa. The highest peak reading during the pedal cycle from all of the examined cycles was considered to be PDAb.

#### Maximal dynamic activity (MxDA)

The peak activation during the pedal cycle (for each muscle), obtained from the 10 s maximal sprint, was considered to be MxDA.

### Statistical analyses

The pre-post reliability of each of the six normalization techniques for each muscle was examined using linear regression, intraclass correlation (ICC) and Cronbach’s alpha analyses. EMG amplitudes obtained from each muscle as a function of time (pre-post) and of the six normalization techniques (technique) were examined statistically using 6 × 2 repeated measures ANOVAs with significance accepted at p<0.05. Post-hoc analyses on the main effects of time and technique were contrasted using pairwise comparisons by means of a Bonferroni adjustment to control type I error. Significant interactions were examined using simple main effects.

## Results

### Reliability

**Figure 1:**
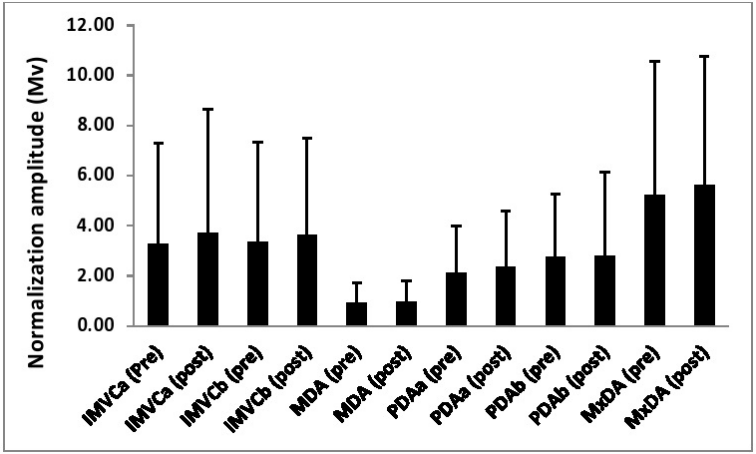
The EMG reference amplitude for the rectus femoris obtained as a function of each normalization technique both pre and post

**Figure 2:**
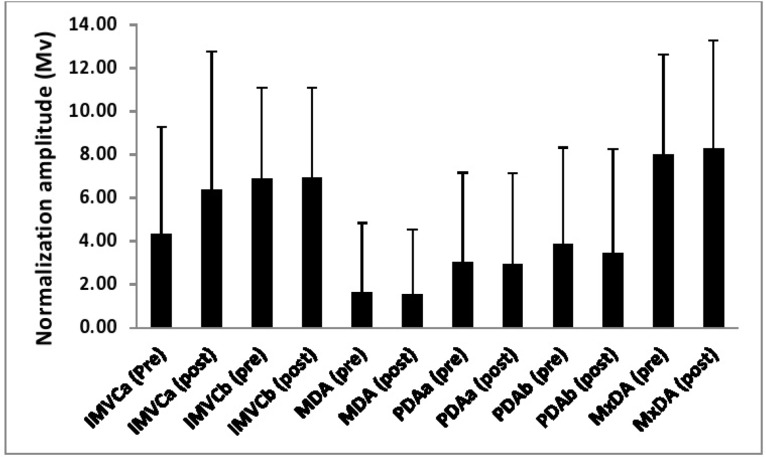
The EMG reference amplitude for the biceps femoris obtained as a function of each normalization technique both pre and post

**Figure 3:**
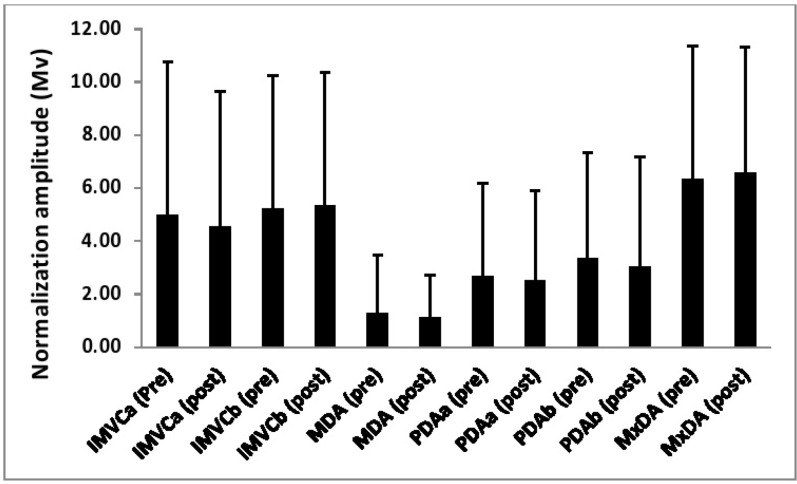
The EMG reference amplitude for the gastrocnemius obtained as a function of each normalization technique both pre and post

**Figure 4:**
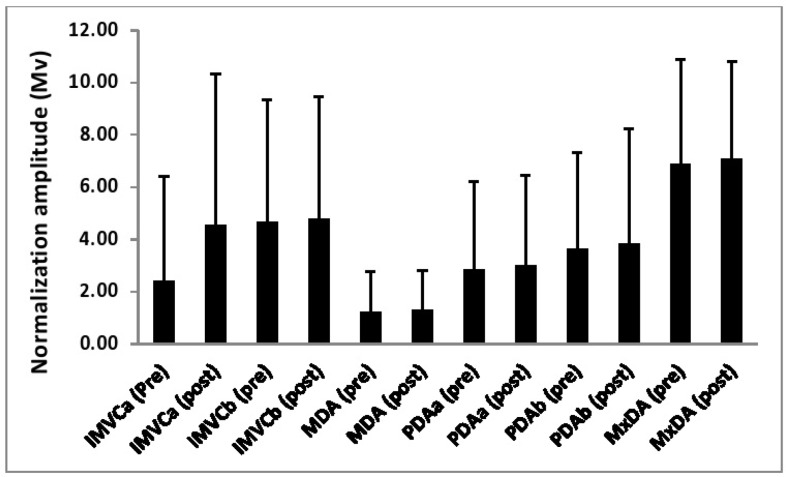
The EMG reference amplitude for tibialis anterior obtained as a function of each normalization technique both pre and post

Tables 1–3 and Figures 1–4 show both the reliability of each normalization technique and the differences in the EMG amplitude as a function of the different methods for each of the examined muscles.

### Rectus femoris

A significant main effect (p<0.01, η^2^= 0.22) was observed for the magnitude of the normalization amplitude as a function of normalization technique. Post-hoc pairwise comparisons showed that a mean amplitude from the MDA technique was significantly (p<0.05) lower than each of the remaining five techniques. Furthermore, it was also shown that the amplitude in the MxDA technique was significantly (p<0.05) greater than the PDAa method.

Significant main effects were observed for the magnitude of the normalization amplitude as a function of both normalization technique (p<0.01, η^2^= 0.55) and time (p<0.05, η^2^= 0.18). Furthermore, a significant interaction (p<0.01, η^2^= 0.24) between normalization technique and time was also found. Simple main effects analysis showed that for the IMVCa technique there was a significant difference (p<0.05, η^2^= 0.25) between pre and post amplitudes.

### Gastrocnemius

A significant main effect (p<0.01, η^2^= 0.32) was observed for the magnitude of the normalization amplitude as a function of normalization technique. Post-hoc pairwise comparisons showed that a mean amplitude from the MDA technique was significantly (p<0.05) lower than each of the remaining five techniques. Furthermore, it was also shown that the amplitude in the MxDA technique was significantly (p<0.05) greater than the PDAa method.

### Tibialis anterior

Significant main effects were noted for the magnitude of normalization amplitude for both normalization technique (p<0.01, η^2^= 0.26) and time (p<0.05, η^2^= 0.22). A significant interaction (p<0.01, η^2^= 0.19) between normalization technique and time was also observed. Further analysis using simple main effects indicated that for the IMVCa technique there was a significant difference (p<0.05, η^2^= 0.21) between pre and post amplitudes.

## Discussion

The aim of the current study was to investigate the reliability of different EMG normalization techniques and their application to cycling analyses. This represents the first attempt to examine the effectiveness of these techniques for cycling. A study of this nature may be of both practical and clinical significance to researchers utilizing EMG in cycling analyses who require a meaningful method of EMG normalization.

The common consensus regarding EMG normalization is that an appropriate reference technique to which the dynamic EMG signals are applied needs to have high levels of repeatability. EMG normalization methods should therefore produce similar results over different testing trials. In support of our hypothesis the reliability analysis showed that the PDAa method was habitually the most reliable normalization technique across all muscles. This concurs with the findings of Ball and Scurr (2011) and Sinclair et al. (2012). With regard to the IMVC techniques the results show that these exhibited much lower reliability. It is in line with previous investigations examining normalization reliability in other sports/ movements (Ball and Scurr, 2011; Sinclair et al., 2012), but disagrees with those of Bolgla and Uhl (2007). It is believed that maximal MVCs may increase activation variability by recruiting a larger number of type II muscle fibers than sub-maximal normalization techniques. Such low levels of reliability for IMVCs are particularly concerning given the widespread utilization of isometric normalization methods, and potentially question the efficacy of analyses using these techniques for the muscles examined in the current study.

Whilst there appears strong evidence that using the dynamic normalization is associated with greater reliability for cycling in relation to other normalization methods there has also been concern that such approaches tend to generate a normal EMG profile for a specific movement. This may eliminate some of the true biological variation from within a participant group (Allison et al., 1993; Knutson et al., 1994). The amount of muscle activation necessary to produce a pre-set power output would differ according to cycling ability and strength. The normalization reference amplitude, obtained from dynamic normalization methods specific to the task under observation, is clearly relative to the task itself and not the maximum capacity of the muscle. This therefore makes comparisons between muscle activity, tasks or individuals difficult. Dynamic task specific methods can however be used to contrast patterns of muscle activation between individuals over time (Bolgla and Uhl, 2007). This may be particularly concerning in clinical analyses using EMG, as the extent of the muscles activation cannot be related to any physiologically relevant parameter. Participants inability to actively contract specific muscles due to pain inhibition and altered neuromuscular function may not be detected (Benoit et al., 2003). As such, there is still concern regarding the homogeneity of the task-specific EMG signal even when reliable normalization techniques are employed.

Our second hypothesis was also supported, in that the different techniques produced significantly different magnitudes for normalization. This finding is in agreement with the observations of Sinclair et al. (2012) who showed during running analyses that different normalization techniques significantly influenced the reference EMG amplitudes. The results of this investigation suggest that different normalization techniques can significantly influence the interpretation of the normalized EMG magnitude. As previously stated, within study EMG values are relative to the normalization amplitude, however, in relation to other studies, data is not comparable if different normalization methods are utilized. Therefore, developing normative muscle activation values for specific movements is problematic (Sinclair et al., 2012). Furthermore, it appears that different normalization techniques should not be used interchangeably as has been commonplace in past EMG analyses. This may serve to facilitate misinterpretation of the EMG amplitude, and further substantiating the notion that the most appropriate normalization technique is critical to achieve empirically meaningful findings.

As would be expected, the IMVCa, IMVCb and MxDA techniques produced the highest EMG reference amplitudes for all muscles. Many studies of a number of dynamic actions report EMG amplitudes in excess of 100% MVC (Winter, 1996). Dynamic EMG signals that exceed 100% MVC indicate that the normalization technique employed to generate the MVC reference does not determine the muscles maximum activation capacity. This is likely to be the case in the MVC techniques that produce low reference amplitudes, such as the MDA and PDA methods. If the maximum activation capacity in each muscle is not elicited during the normalization procedure, a systematic error may be introduced into the data-set which results in an over estimation of the activation level (Harms-Ringdahl, 1996). This may lead to incorrect interpretation of the muscle activity to perform a specific task. Furthermore, if the activation in different muscles is not referenced to the same normalization intensity in individual muscles, this makes comparison of activity levels between muscles difficult.

A limitation of the present study was the all-male sample which may limit its overall generalizability. Females are known to exhibit different lower extremity kinematics in the coronal and transverse planes during the pedal cycle (Sauer et al., 2007). These differences have predominantly been attributed to gender variations in lower extremity structure. Furthermore, muscle fiber composition has also been shown to exhibit gender differences (Miller et al., 1993), with males exhibiting a greater proportion of type I fibres. Both of these parameters have been shown to influence the resultant EMG amplitude during both static and dynamic muscle actions. Therefore, it is unknown as to whether muscle normalization techniques that are applicable to males are equally applicable in females. This is particularly important to researchers and clinicians who contrast muscle amplitudes between genders during movement tasks. It is therefore recommended so the current investigation is repeated using a female sample.

## Conclusions and practical implications

To our knowledge, the current study represents the first to examine the reliability of normalization methods that are used to quantify muscle activation during cycling. The results show that conventional isometric normalization techniques exhibited the lowest levels of reliability and thus their utilization should be discouraged for the analysis of cycling specific muscles. Furthermore the findings also indicate that the PDAa method exhibited the greatest reliability and thus its utilization is encouraged for cycling analyses. Further work is still required to determine the most empirically meaningful technique that is sensitive to alterations in the workload and would allow impartial comparisons between individuals and muscles.

## Figures and Tables

**Table 1:** Linear regression (R^2^) values between pre-post amplitudes as a function of each normalization technique

	**IMVCa**	**IMVCb**	**MDA**	**PDAa**	**PDAb**	**MxDA**
**TA**	0.260	0.225	0.843	0.947	0.753	0.853
**RF**	0.863	0.731	0.984	0.958	0.483	0.732
**BF**	0.692	0.581	0.996	0.996	0.887	0.901
**GM**	0.401	0.392	0.970	0.966	0.956	0.855

**Table 2:** Intraclass correlation (ICC) values between pre-post amplitudes as a function of each normalization technique

	**IMVCa**	**IMVCb**	**MDA**	**PDAa**	**PDAb**	**MxDA**
**TA**	0.621	0.538	0.959	0.968	0.925	0.765
**RF**	0.958	0.812	0.995	0.980	0.806	0.739
**BF**	0.722	0.606	0.999	0.999	0.978	0.868
**GM**	0.777	0.760	0.956	0.992	0.988	0.830

**Table 3:** Croncach’s alpha (α) values between pre-post amplitudes as a function of each normalization technique

	**IMVCa**	**IMVCb**	**MDA**	**PDAa**	**PDAb**	**MxDA**
**TA**	0.648	0.561	0.957	0.986	0.922	0.755
**RF**	0.953	0.807	0.955	0.983	0.895	0.741
**BF**	0.758	0.637	0.998	0.998	0.969	0.863
**GM**	0.770	0.753	0.967	0.991	0.968	0.816
